# Recent advances on diagnosis and management of childhood asthma and food allergies

**DOI:** 10.1186/1824-7288-39-80

**Published:** 2013-12-27

**Authors:** Dani Hakimeh, Salvatore Tripodi

**Affiliations:** 1 Unfall Krankenhaus Marzan, Berlin, Germany; 2 Department of Paediatrics and Allergology Unit, Sandro Pertini Hospital, Via dei Monti Tiburtini, 389, 00157 Rome, Italy

**Keywords:** Asthma, Food allergies, Environmental risk factors, Parental atopy, Guidelines, Hygiene hypothesis, Infections, Management, Nutrition, Dietary, Prediction, Prevention, Therapy

## Abstract

The epidemic of childhood allergic disorders has been associated to the decline of infectious disease. However, exposure to many triggers (airborne viruses, tobacco smoke, pollution, indoor allergens, etc.) contribute to the disease. Breast feeding practices, nutrition, dietary and obesity also play a multifaceted role in shaping the observed worldwide trends of childhood allergies. Guidelines for treatment are available, but their implementation is suboptimal. Then developed countries are slowing learning integrating the development of suitable guidelines with implementation plans. Awareness, psychosocial and family factors strongly influence asthma and food allergy control. Moreover, monitoring tools are necessary to facilitate self-management. By taking into consideration these and many other pragmatic aspects, national public health programs to control the allergic epidemic have been successful in reducing its impact and trace the need for future research in the area.

## Allergy epidemic and the biodiversity hypothesis

Asthma and allergic rhinitis levels among children experienced a boost in the second half of the 20^th^ century especially [1,2] in developed countries, causing enormous burden for patients and societies [3]. However, the epidemiological heterogeneity at country level is great. In Iceland for example, a cohort of 179 children monitored for allergic manifestations for two decades showed a cumulative prevalence of 29% for asthma during the study period, with a peak prevalence of 28% at age of 4 years [4]. Similarly, the prevalence of asthma in Irish schoolchildren, aged 6–9 moved from 21.7% in 2002 to 23.5% in 2007 [5]. By contrast, in Belarus, in a cross-sectional study conducted in 2010 examined 2606 urban and 2422 rural children aged 6–16 years, the prevalence of asthma was as low as 3.84%. Symptoms of hay fever were also astonishingly rare (2.45%), when compared to the prevalence registered in the rest of Europe [6]. In parallel, dramatic increase of food allergies in the recent 15 yrs released a second wave of the allergic epidemic; however causing factors are still uncertain. In Australia for example, 10% of all infants suffer from a confirmed, IgE-mediated food allergy [7]. Many putative factors were here nominated, such as increased consumption (e.g. seafood allergies) [8], progressive urbanization [7], environmental factors and increased atopic disorders of the parents [9-11] or the increase in sensitization to pollen allergens (e.g. profilins, Bet v 1 and other PR-10 molecules, lipid transfer proteins) which cause the oral allergy syndrome [12]. A recent study on 3800 Finnish children demonstrated how children of allergic parents had a 2-fold higher risk to be sensitized/allergic to food if one and 3-fold risk if both parents suffered from any allergic manifestation [13]. On individual level, other factors including severe atopic eczema, maternal anti-helminthic therapy during pregnancy, birth season and order were suggested to affect the risk of food allergy outbreak in childhood [9,14-16]. For example, children born in autumn/winter had significantly a higher rate of food allergies compared with children born in spring/summer according to recent studies on 835 Australian and 1177 Korean children separately [11,15]. Birth order was inversely related to the risk of food allergy and eczema [16].

Although infections are risk factors of chronic urticaria [17] and other allergic diseases, hygiene and anti-infectious treatments contribute to explain the raising trend of asthma and allergies and they can act even before birth. Maternal anti-helminthic therapy, while protecting the fetus from helminth infection, may facilitate allergy as suggested by a randomized, DBPC study with albendazole on more than 2500 Ugandan women. In this trial, a positive relation between mother’s treatment and increased risk for wheezing and eczema in infancy was found [14]. Gram negative bacteria have been the first category of infectious agents believed to protect from asthma and allergies. The Dutch WHISTLER-birth cohort has recently found that exposure to endotoxin in the first two months of life, in combination with house dust mite and cat allergens, is associated with an increase in the neonatal respiratory compliance. The authors concluded that endotoxin may contribute to the development of a normal lung function and maturity [18]. Not only bacteria, but also some viruses may protect of atopy. In American children up to 8 yrs of age, wild-type varicella zoster infection is associated with less atopic dermatitis (AD) and asthma, a phenomenon explained by suppression of IgE production and allergic sensitization and modified leukocyte subsets [19]. Under the hygiene hypothesis, the so-called “farming effect” is by far the most investigated epidemiological model. A meta-analysis of 39 studies showed a 25% lower asthma prevalence among subjects exposed to farming environment compared with unexposed subjects [20]. However, the basis of a protective effect of farming environment is still not sufficient understood. As proposed in the late nineties, a high microbial turnover at mucosal surfaces and a broad spectrum of microbial contacts in the every-day life may be at the basis of the atopy protecting effect of a traditional lifestyle [21,22] also by exerting an influence at the level of GALT [23,24]. This branch of the hygiene hypothesis, which may be defined as the “biodiversity” hypothesis, has been later supported by studies showing that the diversity of microbial flora early in life is protecting from atopic eczema [25,26] and that the diversity of exposure to bacteria or fungi is an atopy protecting factor in farming children [27]. Accordingly, a cross-sectional study of almost 300 German children has shown that the sole ingestion of the farming milk is linked to less asthma by an increase in T-regulatory cells levels [28]. Similarly, local and systemic immune responses of piglets nursed by their mother on a commercial farm differ from those of isolated piglets fed with formula in a “clean” environment and display stronger regulatory components at mucosa level (CD4(+) CD25(+) Foxp3(+) regulatory T-cell) and higher IgG levels to food proteins at weaning [29]. A low-grade (or the so-called “minimal persistent”) inflammation induced by a farming environment in early childhood may protect against allergic diseases. In 653 children from the PASTURE birth cohort, those with undetectable high sensitive CRP (hsCRP) had increased prevalence of sensitization to inhaled and seasonal allergens [30]. However, the farming environment is showing a wheeze-protective effect also independently from atopy, for example by modifying the susceptibility to wheeze during viral infections [31].

The role of atopic sensitization and viral infections in asthma has been extensively reviewed [32] and investigated [33]. An open question remains the sequential relationship between these two components. A longitudinal study on 285 American children with high risk for allergic diseases and asthma demonstrated that sensitization to aeroallergens increases the risk of wheezing caused by HRV (but not by respiratory syncytial virus). In contrast, viral wheeze did not increase the risk for subsequent allergic sensitization. This study’s conclusion was that allergic sensitization is the one who precedes and might promote viral caused wheezing, and not the other way round [34]. However, other infections are also associated with asthma development. The ISAAC-Phase Two study found that pertussis and measles are associated with symptoms of asthma, rhinoconjunctivitis and eczema in schoolchildren aged 8–12 years, independently from IgE responses [35]. As a part of the same study, an investigation among more than 23,900 schoolchildren aged 8–12 years showed that tuberculosis was associated with asthma, hay fever and eczema symptoms, but not to immunization with BCG [36]. In Latin America not only helminthic infections [37] but a broad set of infections interfere with asthma and its atopic background. By contrast, in 1128 urban children from Salvador-Brazil tested to the 8 most common local pathogens those with 3 or fewer infection markers showed a higher prevalence of sIgE and SPT reactivity compared with those with 4 or more infection markers, but no association of these markers to asthma could be established [38]. The heterogeneity of the conclusions reached by different studies highlights the need of large international studies designed to increase sample heterogeneity and evaluation of local factors to minimize their confounding influence. Anyhow, early prevention is now necessary to avoid further progression of the allergy epidemic. Therefore, screening tests should be optimized to devise appropriate and economic national prevention and treatment strategies [9,39].

Guidelines for treatment are available, but their implementation is suboptimal. Then developed countries are slowing learning integrating the development of suitable guidelines with implementation plans. Awareness, psychosocial and family factors strongly influence asthma and food allergy control. Moreover, monitoring tools are necessary to facilitate self-management. By taking into consideration these and many other pragmatic aspects, national public health programs to control the allergic epidemic have been successful in reducing its impact and trace the need for future research in the area.

## Pollution and tobacco smoke

Traffic-related air pollution (TRAP) and biomass fuels (BMF) play a central role in the asthma epidemic especially in middle and low income countries. In Asia, TRAP and BMF increase the risk of asthma outbreaks, asthma exacerbations, respiratory infections and even COPD [40]. But also in a high income country, such as Canada, the frequency and duration of asthma exacerbations were associated with the levels of air pollution [41]. Domestic usage of BMF for fire, especially in low-income countries, is strongly associated with asthma and other lower respiratory diseases. Raising awareness at community level about this problem can reduce the negative impact caused by BMF [42]. In parallel, campaigns and legislations against tobacco smoke must be reinforced in high income countries, where this risk factor has not been yet defeated. Indeed, the ISAAC-Phase Three study among Maltese children demonstrated that passive and active tobacco smoke, that still is a frequent factor in that country, increased the risk of wheezing, exercise-induced wheezing, asthma, and rhinitis [43]. Furthermore, passive tobacco smoke’s influence is even stronger when it interacts with other risk factors, such as atopic sensitization. Among 486 children from the CCAAPS birth cohort, lung function was impaired at the ages 2, 4 and 7 years in atopic polysensitized girls exposed to tobacco smoke [44]. The effect of tobacco smoke seems to be partially affected also by genetic predisposition. Studies in the Norwegian ECA- and GAIN-cohorts have found that CHRNA3/5 polymorphisms on chromosome 15 are positively associated with bronchial hyperresponsiveness, especially in children exposed to tobacco smoke, no matter if set out in-utero or indoor at 10 yrs of age [45]. Similarly, among 157 children of the ECA-birth cohort, the impact of tobacco smoke exposure and pet keeping on allergy was conditioned by a limitation of CD14 methylation levels in exposed subjects [46]. All these data reinforce the concept that tobacco smoke must be contrasted *at population* level but also that risk factors of susceptibility must be taken into account *at individual* level.

## Obesity, physical exercise and nutrition

The asthma epidemic is not the only one among children of developed and developing countries. A parallel increase in obesity and asthma rates suggests that allergic respiratory disease may be linked to dietary factors and behaviors and that the two conditions may influence each other [47]. Accordingly, a cross-sectional questionnaire-based survey in almost 34,700 Japanese children aged 4–5 years demonstrated how important obesity is in asthma management; current asthma was significantly more prevalent in overweight children compared with underweight and normal weight children after adjusting for other variables, such as gender, other coexisting allergic diseases, and parental history of asthma [48]. Weight reduction has been associated with variations of leptin, ghrelin and obestatin blood concentrations [49] thus correction of obesity implies changes of adipokines supposed to influence allergic inflammation.

The epidemic of childhood allergic disorders has been associated to the decline of infectious disease. However, exposure to many triggers (airborne viruses, tobacco smoke, pollution, indoor allergens, etc.) contribute to the disease. Breast feeding practices, nutrition, dietary and obesity also play a multifaceted role in shaping the observed worldwide trends of childhood allergies.

A smaller study among Italian children has shown that the female gender is a risk factor for asthma associated to obesity [50]. Regular physical exercise may even induce beneficial immune system responses and reduce pro-inflammatory cytokines secretion [51]. In this respect, the positive influence of sport on asthma encourages physical activity in asthmatic children who are obese or overweight [51]. Nevertheless, physical can play a role on FEIA and anaphylaxis [52], hence only physical activities that are well tolerated should be promoted in at risk asthmatic patients.

The impact of nutrition on the development of respiratory disorders in childhood may begin already before birth. In a Finnish population-based birth cohort study (n = 2441), mother’s reduced intake of certain foods (leafy vegetables, malaceous fruits, certain fatty acids and chocolate) during pregnancy was associated with increased risk of wheeze (but not asthma) in her child at 5 years of age [53,54]. However, ‘Western-Diet” including a higher intake of meat, eggs, vegetables’ oil and white vegetables was linked to a reduce risk of wheezing among children aged 16–24 Months [55]. The intake of vitamin D is also increasingly investigated in this area. In an experimental mouse model, vitamin D deficiency *in utero* was found associated with enhanced allergen-induced lymphocyte responses and cytokine production into airways [56]. A case–control study of children with severe therapy-resistant asthma, moderate asthma and healthy controls demonstrated that patients with severe therapy-resistant asthma have significantly lower levels of vitamin D. Decreased levels were also associated with increased airways smooth muscle mass, worse asthma control and lung function [57]. Vitamin D levels may also explain seasonal variations in food allergies risk [15]. Moreover, nutritional deficits have been linked to food allergies [9,58] and supplementary nutrients have been proposed. A recent case–control study on 191 French children (mean age 4 yrs) showed that food allergic patients are smaller than healthy controls [59]. On the other hand, extensive nutrition restrictions in food allergic children are not always necessary and should be established on a case by case fashion, as demonstrated by a recent study on 145 British peanut allergic children [60]. Last but not least, the impact of breastfeeding on asthma and food allergy still remains a hotly debated topic. A longitudinal study on 882 Australian and Swedish children with parental wheeze or asthma found a lower risk for developing asthma at age 4 and 8 yrs among breast-fed, compared to bottle-fed children. Furthermore, breast-feeding did not influence the sensitization rate to airborne allergens but it was a risk factor for sensitization to cow’s milk, peanuts and eggs [61]. Similarly, prolonged breastfeeding and delayed introduction of solid food provided no evidence to be protective from atopic eczema in a population of over 18,700 Taiwan infants [62].

## Prediction and prevention

Suitable and reliable prediction tools are essential to identify targets for prevention. Since many years clinical scientists are trying to find markers predicting asthma. A recent study has shown that inflammatory markers, including many interleukins (IL-2, IL-4, IL-8 and IL-10) are elevated in the exhaled breath condensate already during the pre-clinical phase anticipating wheezing [63]. Furthermore, perinatal factors may affect lung maturity and contribute to cause wheezing and asthma. Neonatal jaundice, for example, increased the risk of asthma in the first six years of life among Taiwanese neonates [64]. Atopic sensitization in infancy has been often used as a predictor of atopic diseases at school age. Recently, Item Response Theory (IRT) models, have been applied to SPT results at 2 years of age to predict persistent wheeze, rhino-conjunctivitis, and eczema at age 4 and to identify the allergens most associated with atopy predisposition [65].

Prediction models are used to facilitate the identification of targets for prevention. Until a few years ago, allergic asthma prevention has been mostly based on allergen avoidance. Studies results though show no or little benefit from allergen avoidance. By contrast, a national plan for allergy and asthma prevention, recently implemented in Finland, has shown that implementation of balanced diets, physical activity and protection from other allergy promoting factors such as tobacco smoke represent adequate prevention methods [66]. This Finnish program was then based on a multifaceted strategy. The complexity of the immune response and of allergy pathogenesis is making more and more questionable if one kind of intervention alone is sufficient to prevent allergic disease in high risk children. More and more relevance is being given to the concept of a poly-factorial intervention, based on both, avoiding risk factors and providing protective factors. Unfortunately, the feasibility of interventions in everyday life declines with their increasing complexity [66-68]. For example, a study on 476 Dutch children demonstrated that PREVASC multifaceted intervention was efficient in reducing the exposure to inhalant and food allergens. Nevertheless, this program was unable to reduce the prevalence of asthma at the age of 6 yrs, as the parents were unable to properly implement all the measures of this complex prevention plan [69].

In contrast to asthma, studies on prediction and prevention of atopic eczema are still providing relatively conflicting outcomes. However, the “classical” biomarkers of allergy, such as total and specific IgE levels are still considered valuable pre-clinical predictive biomarkers [70-73]. For example, elevated maternal IgE levels during pregnancy were associated in non-atopic mothers to increased risk of eczema in offspring [70]. Elevated cord blood IgE, together with specific LT-α and FcϵRI-β genotypes and a higher maternal stress during pregnancy were linked to a higher risk of eczema at age of 2 yrs [71]. Similarly, not SPT [72] but IgE levels can be useful in predicting a positive food challenge test [73]. Although these biomarkers can help predicting atopic eczema [74], prevention is unfortunately still difficult. Probiotics are widely marketed as useful against food allergy [75,76], respiratory infections [77] and airway inflammation [78], but their preventive efficacy has not been confirmed. For example, Lactobacillus rhamnosus GG, given to the mother during pregnancy, had no influence on her child’s gut colonization profile [79] and their content in cow’s milk and egg’s white proteins may be even dangerous for allergic patients [80]. However, supplementation of the probiotic Lactobacillus plantarum CJLP133 provided beneficial results in the treatment of pediatric AD in a case–control study on 88 Korean children between 12 months and 13 yrs [81].

## Monitoring and control

Asthma control is essential to prevent long-term consequences of asthma and improve patients’ quality of life. Hence, many studies focus on identification of reliable diagnostic tools to monitor asthma control. While the diagnostic role of atopic tests, lung function and challenge tests in asthma are quite established since decades that of breath condensate is still hotly debated. Exhaled nitric oxide (NO), a non-invasive marker of airway inflammation, can be implemented in the diagnosis and management of asthma and its strong relation to atopy, (e.g. house dust mite sensitization) has been recently described [82]. Furthermore, elevated bronchial NO output is associated with an increased risk of exercise-induced bronchoconstriction in atopic patients during childhood and adolescence [51]. Similarly, fractional exhaled NO (FeNO) was also elevated in the sputum of asthmatics with coexisting food allergy suggesting an association of asthma with food allergies through an increased eosinophilic inflammation [83]. Accordingly, in 467 children from the European Community Respiratory Health Survey II, FeNO was only elevated in asthmatic patient with simultaneous sensitization to food and pollen allergens [84]. Unfortunately, FeNO levels in exhaled breath may be not always appropriate as a marker to monitor asthma control over time [85]. This has been also confirmed by a study showing a poor accuracy to discriminate well- from not well-controlled asthma in a case–control study among 664 Dutch children aged 4–12 yrs [86].

Other measures of oxidative stress have been also tested. A case–control study on 219 Turkish children has shown that higher concentrations of Malondialdehyde and GSH in exhaled breath condensate are a marker of asthma and allergic rhinitis and are associated with enhanced oxidative stress [87]. Another case–control study among 22 asthmatic Swedish children (6–18 years) demonstrated the relevance of basophil allergen threshold sensitivity (also CD-sens) in identifying those with the most severe and uncontrolled allergic asthma [88]. Last, clinical indexes are also intensively investigated, as they can allow daily monitoring. The visual analogue scale (VAS), a widespread assessment in Pediatrics, confirmed its usefulness in measuring breathlessness perception in Italian asthmatic children and was significantly related to lung function [89]. However, other clinic assessments lack so far of unsatisfying accuracy and reliability levels. A study in 314 patients has recently shown that both, GINA-guided and C-ACT-guided (Childhood-Asthma Control Test) assessment have a poor accuracy and reliability in measuring the level of asthma control, if used separately [90].

Anti-inflammatory drugs and bronchodilators [91-93] and antibiotics (e.g. macrolides) [94] help controlling asthma but allergen-specific immunotherapy (SIT) is the only treatment potentially changing the natural history of the allergic component of moderate asthma, with a potential long lasting exacerbation-preventing effect and corticosteroid-sparing effect [95]. However, due to the difficulty in carrying out placebo controlled trials, efficacy of SIT, especially in its subcutaneous version (SCIT), has been so far demonstrated in children by a few trials only [96-98].

Food allergy monitoring and control is a highly developing area of investigation and we can consider here only a few examples of studies dealing with the need of molecular diagnosis, the antigen-specificity of oral tolerance induction and the use of epinephrine, respectively.

Cross-reactivity and pan-allergens represent one of the difficulties that often hamper the diagnosis of allergies to vegetal food in childhood. Therefore, diagnostic tools should be developed to support clinicians in distinguishing between tolerant and allergic and then between primary and secondary allergic patients. In birch-pollen-associated secondary soy allergy, SPT with soy flour and molecular diagnostic of specific IgE to Gly m4 facilitate a correct diagnosis [99]. IgE levels to seed storage proteins Ara h1, Ara h2 and Ara h3 allow distinguishing peanut-allergic from tolerant patients [100,101]. By contrast, peanut-specific IgG or IgG4 levels were elevated in peanut-sensitized children and did not indicate tolerance or protection from sensitization as observed in other atopic diseases [102]. Similar findings were also shown in patients with egg allergy, where elevated levels of specific IgG to ovalbumin, β-lactoglobulin and α-casein were associated with positive SPT to egg. In contrary, elevated IgG4 levels to α-casein was linked to a negative DBPC cow’s milk challenge [103]. On the other hand, cytokines and other immunological markers are being investigated for a more precise monitoring of food allergies. For example, the interleukin IL-21 may be involved in the pathogenesis of atopic dermatitis [104] and the chemokine CCL18, also known as pulmonary and activation-regulated chemokine (PARC), may be linked to disease severity [105].

Studies on oral tolerance induction (OIT) are defining more and more the usefulness and limitations of this novel therapeutic approach. Cow’s milk allergy affects 1.9-4.9% of the children, of which a few might suffer from a protein energy malnutrition caused by the subsequent dietary [106]. Therefore, oral tolerance induction provides a chance for these patients to avoid possible late-implication caused by dietary restrictions such as growth impairment. Recent studies on SOTI demonstrated an increase of regulatory cells such as Foxp3+ regulatory T-cells and other hypo-proliferative subset of T-cells (CD4+, CD38+ and CD45RO-) which characterize tolerance development, suggesting possible pathways for tolerance induction in cow’s milk and hen’s egg allergic patients [107,108]. The induction of clinical tolerance, however, is antigen specific. This aspect is essential from a clinical stand point, as many children with cow’s milk allergy react also to goat’s or sheep’s milk. Unfortunately, orally induced tolerance to cow’s milk does not protect these children from reactions to the milk of other mammals [109,110] and elimination diets are sometime not sufficient to avoid severe reactions in allergic children [111]. A few children with severe food allergies cannot be treated with SOTI, as they do not tolerate even minute amount of the relevant allergen. In these children, proper education, availability and correct use of epinephrine is often essential. Unfortunately this is not always the case. A recent investigation of 1361 adverse reactions to food in the US showed that only one third of the reactions requiring intervention with epinephrine were correctly managed [58]. Actually, guidelines on anaphylaxis are not sufficiently implemented although they are distributed to most pediatricians [112]. Education and awareness about anaphylaxis is therefore still insufficient to guarantee guidelines implementation and the guidelines themselves should be perhaps simplified and made more user-friendly [113].

## Psychological and public health aspects

Despite intensive research and progress, allergic diseases in childhood still represent a complex challenge for the family and the society. They has a considerable impact on quality of life for the patients and a financial burden for the society caused by increased costs, morbidity and mortality [114]. A recent meta-analysis considering almost 3550 children with asthma has shown that patients suffer from more depression and anxiety, independently from the level of severity of their asthma [115]. Similarly, among the asthmatic adults screened in the Asthma Insight and Management (AIM) survey in the United States, patients rated their health as “only fair,” “poor,” or “very poor,” or experienced limitations in activity because of health problems, compared with the general population. This indicates that the burden of asthma remains high in the US despite the availability of updated treatment guidelines and new therapies [116]. Moreover, severe reactions observed in food allergies have also immense impact on patients’ quality of life. A recent survey on the life quality of food allergic children showed that patients who experienced severe systemic reactions, with multiple food allergies or with siblings/mother affected by allergies score worse in quality of life questionnaire [117]. In this respect, it is interesting to note that a farming environment, which in somehow implies a healthier contact with nature, can influence not only physical and objective reactions but also the psychological well-being of allergic and asthmatic patients [118].

Hence, not only for the patients, but also for their families it is important to establish clear plans beyond the patient’s direct needs by involving the community. Such perspectives can be successfully achieved by national-wide management programs aimed at minimizing disease’s impact on the whole society and the healthcare systems [66]. Further inputs provided by parents and/or patients associations might be useful. A management curriculum for the parents of newly diagnosed food allergic children can reduce both stress and anxiety and improve the quality of life of the allergic children and their parents [119].

Guidelines for treatment are available, but their implementation is suboptimal. Developed countries are slowing learning integrating the development of suitable guidelines with implementation plans. Awareness, psychosocial and family factors strongly influence asthma and food allergy control. Moreover, monitoring tools are necessary to facilitate self-management. By taking into consideration these and many other pragmatic aspects, national public health programs to control the allergic epidemic have been successful in reducing its impact and trace the need for future research in the area.

Moreover, awareness for food allergies and anaphylaxis must be improved also among teachers and school-personnel, since schools are the places with the highest attack-related death rate [120]. Recommendations must not only guide diagnosis and treatment, but should also prevent unjustified and exaggerated allergen avoidance, which heavily affect life quality and immunologic tolerance development. This is true not only for food, but also for respiratory allergies. Accordingly, asthma control programs have to be made easy and integrated into everyday life. An authentic reflection of the difficulties facing implementation of asthma control programs in real life has been shown by the results of the CHOICE survey on 1000 American asthmatic patients. Almost the half of the patients did not use controller medications while 80% of those had a persistent asthma. Moreover, only 14.3% of patients on controllers were well controlled [121]. Other studies reported high levels of over- and under-treatment for asthma [122,123]. Hence, despite updated guidelines, the large offer in medication and active national management programs, control programs are still not well implemented in “real life”. Old guidelines included also unnecessary recommendations: for example, pet avoidance has been a long-lasting prescription for asthma prevention. Recent studies have confirmed that pet exposure in early life is not to be suspended even in families with “at risk” atopic/allergic children, unless pet already cause symptoms [124]. Guidelines should be therefore “user friendly”, so that GPs, parents, health care centers and schools can easily learn how to handle asthma without useless restriction or even harming influences for the affected children. This has been confirmed by a recent study on 170 Dutch children, where parental concerns about adverse consequences of the medication and low maternal education were associated with uncontrolled asthma [125]. Attention to guidelines feasibility has probably greatly contributed to the success of the Finnish plan for asthma control [66]. The Finnish experience can therefore encourage other countries to in their attempt to revert the epidemic trend of asthma and allergies or at least to control better the social impact of this childhood chronic disease.

## Conclusion

The most recent advances in the field of childhood asthma summarized in this review give us an idea of how fast clinical research in this area is making progress. Solid approaches to avoid risk factors and to promote protective factors should be established according to the recent findings. Nevertheless, many studies demonstrated the huge lack in adopting and implementing recommendations and guidelines in everyday life, which require further development of current intervention and control strategies in the framework of national asthma control programs to choke the worldwide asthma epidemic.

## Abbreviations

AR: Allergic rhinitis; BMI: Body mass index; CI: Confidence interval; DBPC: Double blinded placebo controlled; GINA: Global initiative for asthma; HDM: House dust mites; HRV: Human rhinovirus; ISAAC: International study of asthma and allergies in childhood; OR: Odds ratio; PAH: Polycyclic aromatic hydrocarbons; PM: Fine particular matter; RSV: Respiratory syncytial virus; SCIT: Subcutaneous specific immunotherapy; SLIT: Sublingual specific immunotherapy; SOTI: Specific oral tolerance induction; SPT: Skin prick test; Yr(s): Year(s).

## Competing interests

The authors declare that they have no competing interests.

## Authors’ contributions

DH wrote the draft version and made the literature search. ST revised the final version. Both authors read and approved the final manuscript.
